# Foxg1-Cre Mediated Lrp2 Inactivation in the Developing Mouse Neural Retina, Ciliary and Retinal Pigment Epithelia Models Congenital High Myopia

**DOI:** 10.1371/journal.pone.0129518

**Published:** 2015-06-24

**Authors:** Olivier Cases, Antoine Joseph, Antoine Obry, Mathieu D. Santin, Sirine Ben-Yacoub, Michel Pâques, Sabine Amsellem-Levera, Ana Bribian, Manuel Simonutti, Sébastien Augustin, Thomas Debeir, José Alain Sahel, Annabel Christ, Fernando de Castro, Stéphane Lehéricy, Pascal Cosette, Renata Kozyraki

**Affiliations:** 1 INSERM, U968, Paris, F-75012, France; 2 UPMC Univ Paris 06, UMR_S968, Institut de la Vision, Paris, F-75012, France; 3 CNRS, UMR_7210, Paris, F-75012, France; 4 CNRS, UMR_6270, PISSARO Proteomics Platform, Institute for Research and Innovation in Biomedicine, Rouen University Hospital, Rouen, F-76821, France; 5 INSERM, U905, PISSARO Proteomics Platform, Institute for Research and Innovation in Biomedicine, Rouen University Hospital, Rouen, F-76821, France; 6 Centre de NeuroImagerie de Recherche, Paris, F-75013, France; 7 Centre Hospitalier National d’Ophthalmologie des Quinze-Vingts, INSERM-DHOS CIC 503, Paris, F-75012, France; 8 Grupo de Neurobiologia del Desarollo-GNDe, Hospital Nacional de Parapléjicos, Toledo, Spain; 9 Sanofi-Ophthalmology, Paris, F-75012, France; 10 Max-Delbrück-Center for Molecular Medicine, Berlin, D-13125, Germany; Massachusetts Eye & Ear Infirmary, Harvard Medical School, UNITED STATES

## Abstract

Myopia is a common ocular disorder generally due to increased axial length of the eye-globe. Its extreme form high myopia (HM) is a multifactorial disease leading to retinal and scleral damage, visual impairment or loss and is an important health issue. Mutations in the endocytic receptor *LRP2* gene result in Donnai-Barrow (DBS) and Stickler syndromes, both characterized by HM. To clearly establish the link between *Lrp2* and congenital HM we inactivated *Lrp2* in the mouse forebrain including the neural retina and the retinal and ciliary pigment epithelia. High resolution *in vivo* MRI imaging and ophthalmological analyses showed that the adult *Lrp2*-deficient eyes were 40% longer than the control ones mainly due to an excessive elongation of the vitreal chamber. They had an apparently normal intraocular pressure and developed chorioretinal atrophy and posterior scleral staphyloma features reminiscent of human myopic retinopathy. Immunomorphological and ultrastructural analyses showed that increased eye lengthening was first observed by post-natal day 5 (P5) and that it was accompanied by a rapid decrease of the bipolar, photoreceptor and retinal ganglion cells, and eventually the optic nerve axons. It was followed by scleral thinning and collagen fiber disorganization, essentially in the posterior pole. We conclude that the function of LRP2 in the ocular tissues is necessary for normal eye growth and that the *Lrp2*-deficient eyes provide a unique tool to further study human HM.

## Introduction

Myopia usually results from an eye that has become too long, generally through elongation of the vitreous chamber. It is a major health issue because of its increasing prevalence worldwide and the vision-threatening pathologies associated with high myopia (HM) [1,2]. HM affects 1%-3% of the population and is defined as refractive error greater than -6 diopters with axial eye length more than 26 mm [3,4]. HM has a clearly defined genetic component and both isolated and syndromic forms have been described [5,6]. In addition to the genetic mutations that influence the size of the eye the clinical state of the highly myopic globe also depends on age and exposure to environmental factors. Indeed highly myopic eyes are especially susceptible to develop degenerative changes including myopic chorioretinal atrophy and posterior staphyloma, complications that can cause visual loss [2,7–9]. It is currently difficult to control the progression of HM, to prevent or treat its associated complications and further comprehension of its pathogenesis is hampered by the lack of a genetic animal model that mimics the human pathology [10].

Mutations in the *LRP2* gene (2q23.3–3.1) encoding the multiligand endocytic receptor LRP2/Megalin have been associated with two distinct rare genetic syndromes, the Donnai-Barrow or facio-acoustico-renal syndrome (DBS/FOAR) and the Stickler syndrome characterized by facial, skeletal and auditory findings [11,12]. Ocular defects, including HM, are common in these syndromes suggesting that *LRP2* might be involved in the regulation of eye growth. Recently nonsense mutations in the zebrafish *lrp2* were shown to result in uni- or bi-laterally enlarged eyes (*bugeye*) and increased intra-ocular pressure [13]. *Bugeye* may be an interesting tool to study adult-onset glaucoma-associated phenotypes including myopia [13,14], nevertheless the eyes of fish are very different anatomically and optically compared with mammals and studies involving higher vertebrates may be more relevant for high myopia associated pathologies [15]. More recently, the inactivation of the *Lrp2* gene in the mouse epiblast [16,17] *via* the *More-Cre* strain was reported to also result in adult-onset myopia [18] but the authors do not provide any evidence that the *More-Cre Lrp2* mutants indeed model human myopia. A model summarizing traits of HM and its associated complications is thus still missing. Moreover there is currently no information on the tissue autonomy of the ocular *Lrp2*-loss of function phenotype.

We here use a floxed *Lrp2* allele [19] and the *Foxg1-Cre* transgene [20], to inactivate *Lrp2* in the developing ocular structures. Homozygous *FoxG1*.*Cre Lrp2* ^*L/L*^ *(Lrp2* ^*FoxG1*.*cre-KO*^) mutants display early, post-natal onset bilateral eye enlargement principally due to increased vitreous chamber depth. Whereas the intraocular pressure is apparently normal in the mutants, degenerative changes are evidenced in the retina and sclera, including formation of posterior staphyloma. In addition to show that *Lrp2* ^*FoxG1*.*cre-KO*^ mice model congenital HM our study also indicates that LRP2 is required in the ocular tissues for normal eye growth. Because age-associated exposure to environmental factors influences the progression of HM [2,21] and the ocular phenotype of the mutants progressively worsens we propose that the *Lrp2* ^*FoxG1*.*cre-KO*^ mouse will provide a valuable tool to study the contribution of aging in the evolution of HM.

## Results

### Excessive bi-lateral eye enlargement in Lrp2^FoxG1.cre-KO^mutants

**Fig 1:**
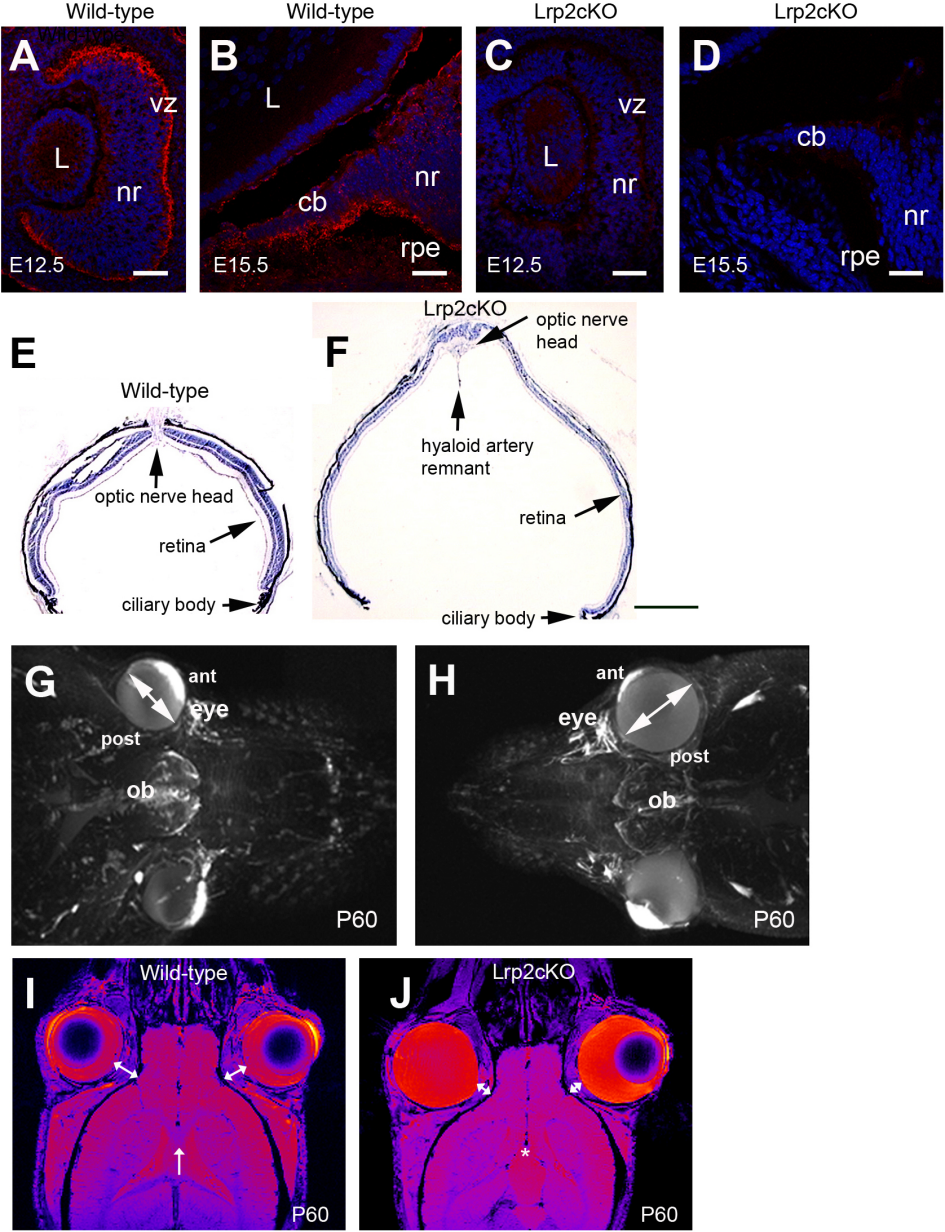
Lrp2-deficient eyes are abnormally enlarged. Sagittal cryosections through the developing eye (**A-D**). Lrp2 is expressed in the developing neuroretina (nr) at E12.5 (**A**). From E15.5 onward the signal is restricted in the lens (L) facing inner layer of the ciliary body (cb) epithelium, a low expression is also seen in the outer layer of the CB and in the retinal pigmented epithelium (rpe) (**B**). Loss of Lrp2 signal in *Lrp2* ^*FoxG1*.*cre-KO*^ mutants at E12.5 and E15.5 (**C, D**). Sagittal cryosections of control and mutant retinas at P60 showing retinal thinning and the presence of a posterior staphyloma in the mutant (**E**, **F**). Reconstruction of the mouse face using MRI at P60 (**G**, **H**). The *Lrp2* ^*FoxG1*.*cre-KO*^ mutants display bilateral eye enlargement, through the anterior-posterior axis and the equatorial diameter (double headed arrows in **G, H**). Horizontal MRI images showed that the retrobulbar space, between the orbit and the eyeball, (double-headed arrows in **I, J**) is decreased in *Lrp2* ^*FoxG1*.*cre-KO*^ mutants. The corpus callosum (arrow in **I**) is not formed in the mutants (asterisk in J). (vz) ventricular zone. Scale bars: 25 μm in A-D; 600 μm in E, F.

Lrp2 is expressed in the eye primordium by E9.0 [22], as soon as the optic vesicle forms. Within the optic cup LRP2 displays a high-to-low dorsal-to-ventral gradient in the presumptive neural retina and retinal pigment epithelium (Fig 1A). After E15.5 the LRP2 signal is concentrated at the inner, nonpigmented, layer of the ciliary body epithelium whereas a low signal is also found at the outer, pigmented, layer of the ciliary body epithelium and the retinal pigment epithelium (Fig 1B). Other Lrp2 expressing tissues during eye development include the cephalic neural crest cells [23] and the optic nerve astrocytes [24]. Conditional inactivation of *Lrp2* in the neural crest, via *Wnt1-Cre*, or in the astrocytes, via *GFAP-Cre*, did not alter retinal differentiation as shown in S1 Fig, or eye formation indicating that *Lrp2* expressed in these tissues was not critical for eye morphogenesis.

To inactivate *Lrp2* in the forebrain we used the *FoxG1*.*Cre* deleterious mouse strain active from E9.0 onward [20]. Complete *Lrp2* ablation within the neural retina and the developing ciliary body was efficient by E12.5 (Fig 1C and 1D). Homozygous *Lrp2* ^*FoxG1*.*cre-KO*^ mutant mice were viable and showed a striking bi-lateral eye enlargement (Fig 1E and 1F). High-resolution small animal magnetic resonance imaging (MRI) revealed that the excessive eye enlargement was associated with a shorter inter-ocular distance and that the retrobulbar space; i.e. the space between the eyeball and the orbit, was reduced in the mutant eyes (Fig 1G–1J). In addition to the ocular phenotype the *Lrp2* ^*FoxG1*.*cre-KO*^mutants lacked a corpus callosum (arrow and asterisk in Fig 1I and 1J respectively), a feature reminiscent of the human DBS phenotype [11].

### Excessive axial elongation and posterior pole degenerative changes in the Lrp2^FoxG1.cre-KO^ mutant eyes

**Fig 2:**
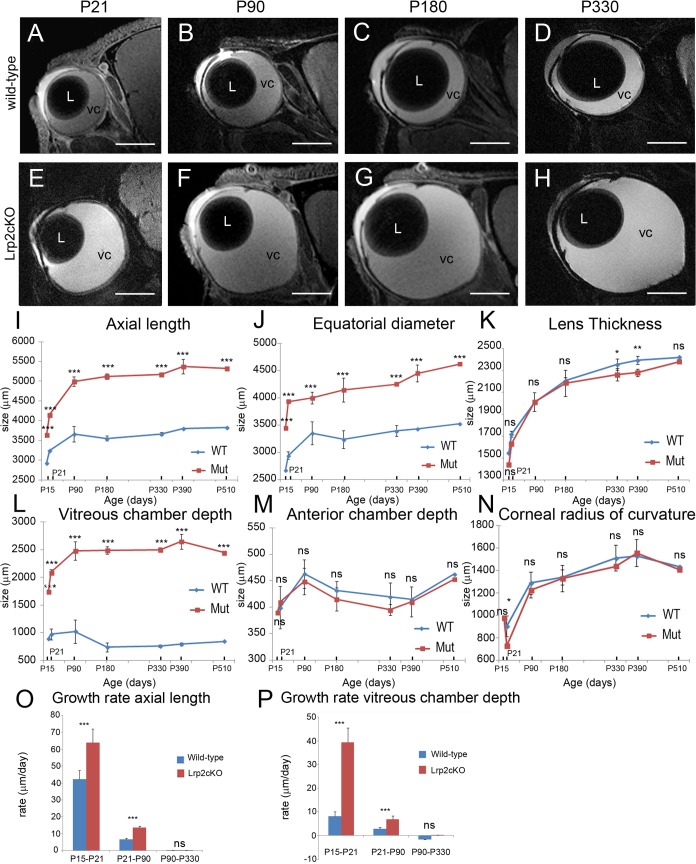
MRI analysis of post-natal eye growth in control and *Lrp2* ^*FoxG1*.*cre-KO*^ mutants over the first year of life. High resolution sagittal slices through the optical axis of the right eye of each mouse were acquired (**A-H**). A variety of optical parameters were extracted from the MRI images collected from groups of control and mutant mice at the ages indicated (**I-N**). Growth rate of axial length and vitreous chamber depth (**O**, **P**). A two-way ANOVA post hoc Tukey test was used, *P*<0.05, **P<0.01, ***P<0.001, ns: not statistically significant, values are mean ± SEM of 4 animals per age and genotype. Scale bars: 1500 μm in A-H.

To further characterize the mutant ocular phenotype we used the 11.7T MRI system to conduct longitudinal studies on control and mutant littermates from P15 onward (Fig 2A–2N). Sequential, virtual sagittal slices through the optical axis of the eye of each mouse were acquired and used to extract various ocular parameters [25] including axial length (AL), equatorial diameter (ED), lens thickness (LT), vitreous chamber depth (VCD), anterior chamber depth (ACD) and corneal radius of curvature (CRC). AL and ED increased in both genotypes between P15 and P510 and were significantly different between control and mutant littermates at all the ages analyzed (two–way ANOVA: AL, main effect “genotype”: *F*_(1, 42)_ = 4502, *P* < 0.0001, interaction: *F*_(6, 42)_ = 33.13, *P* < 0.0001, Fig 2I; ED, main effect “genotype”: *F*_(1, 42)_ = 11080, *P* < 0.0001, interaction *F*_(6, 42)_ = 61.43, *P* < 0.0001, Fig 2J, n = 4 animals per age and genotype). Indeed, AL and ED remained dramatically higher in *Lrp2*-deficient eyes throughout the study (two-way ANOVA followed by post hoc Tukey test, all ages ***P<0.001). As a consequence, biometric parameters depending on AL and ED such as asphericity coefficient (AL/ED), circumference and area remained also significantly higher in the *Lrp2* ^*FoxG1*.*cre-KO*^ mutants (two-way ANOVA: AL/ED, main effect “genotype”: *F*_(1, 42)_ = 489, *P* < 0.0001, interaction: *F*_(6, 42)_ = 65.86, *P* < 0.0001, Table A in S2 Fig; circumference, *F*_(1, 42)_ = 5090, *P* < 0.0001, interaction: *F*_(6, 42)_ = 50.63, *P* < 0.0001, Table B in S2 Fig, area, *F*_(1, 42)_ = 9020, *P* < 0.0001, interaction: *F*_(6, 42)_ = 29.45, *P* < 0.0001, Table C in S2 Fig; n = 4 animals per age and genotype). The LT increased during postnatal development of both control and *Lrp2*-deficient eyes, and was different between the two genotypes (main effect “genotype”: *F*_(1, 42)_ = 13.65, *P* = 0.0006, interaction: *F*_(6, 42)_ = 3.597, *P* = 0.0057, n = 4 animals per age and genotype, Fig 2K). The LT of the *Lrp2*-deficient eyes was significantly lower at P330 and P390 (two-way ANOVA followed by post hoc Tukey test, *P = 0.0255 < 0.05 and **P = 0.0058 < 0.01, respectively). In control eyes, the VCD was the only AL component that declined during postnatal development. In contrast, the VCD of the *Lrp2*-deficient eyes increased dramatically at all ages studied (two-way ANOVA, main effect “genotype”: *F*_(1, 42)_ = 19630, *P* < 0.0001, interaction: *F*_(6, 42)_ = 155.5, *P* < 0.0001, all ages studied ***P<0.001 between the two genotypes, n = 4 animals per age and genotype, Fig 2L). The ACD increased similarly in both genotypes from P15 to P510 (main effect “genotype”: *F*_(1, 42)_ = 0.7372, *P* = 0.3962, interaction: *F*_(6, 42)_ = 1.861, *P* = 0.1256, n = 4 animals per age and genotype, Fig 2M) and so did the CRC (main effect “genotype”: *F*_(1, 42)_ = 2.082, *P* = 0.1565, interaction: *F*_(6, 42)_ = 1.153, *P* = 0.3495, n = 4 animals per age and genotype, Fig 2N).

To analyze the growth rates of AL and VCD biometric parameters, we distinguished three different phases; phase 1 (P15-P21), phase 2 (P21-P90) and phase 3 (P90-P330) (Fig 2O and 2P). Phase 1 was characterized by a very rapid enlargement of the eyeball, in both control and mutant mice; the AL growth rate was however higher in the mutant (64 μm/d) than in the control eyes (42.4 μm/d). During phase 2 the mean growth rate dropped to 13.4 μm/d in the mutants and 6.5 μm/d in the controls and further slowed to 0.3 μm/d and 0.02 μm/d respectively in phase 3. The very rapid increase in AL during phase 1 was associated with a higher VCD growth rate in the mutants (39.4 μm/d) compared with the controls (8.1 μm/d). In phase 2 the VCD mean growth rate decelerated in both control and *Lrp2* ^*FoxG1*.*cre-KO*^ eyes but remained two-fold higher in the mutants; in phase 3 a very slow increase in VCD (0.07 μm/d) was still recorded exclusively in the mutants (two-way ANOVA, AL: genotype effect: *F*_(1, 18)_ = 96.98, *P* < 0.0001; period effect, *F*_(2, 18)_ = 1151, *P* < 0.0001; interaction: *F*_(2, 18)_ = 36.54, *P* < 0.0001. VCD: genotype effect, F_(1, 18)_ = 1631, P < 0.0001; period effect, *F*_(2, 18)_ = 2371, *P* < 0.0001; interaction: *F*_(2, 18)_ = 943.4, *P* < 0.0001, n = 4 animals per period and genotype, Fig 2O and 2P). In agreement with early onset and continuous extension of the ocular axis posterior staphyloma could systematically be evidenced from P21 onward on MRI sections of the mutant eyes (Fig 2E–2H) as well as on histological sections (Fig 1F).

**Fig 3:**
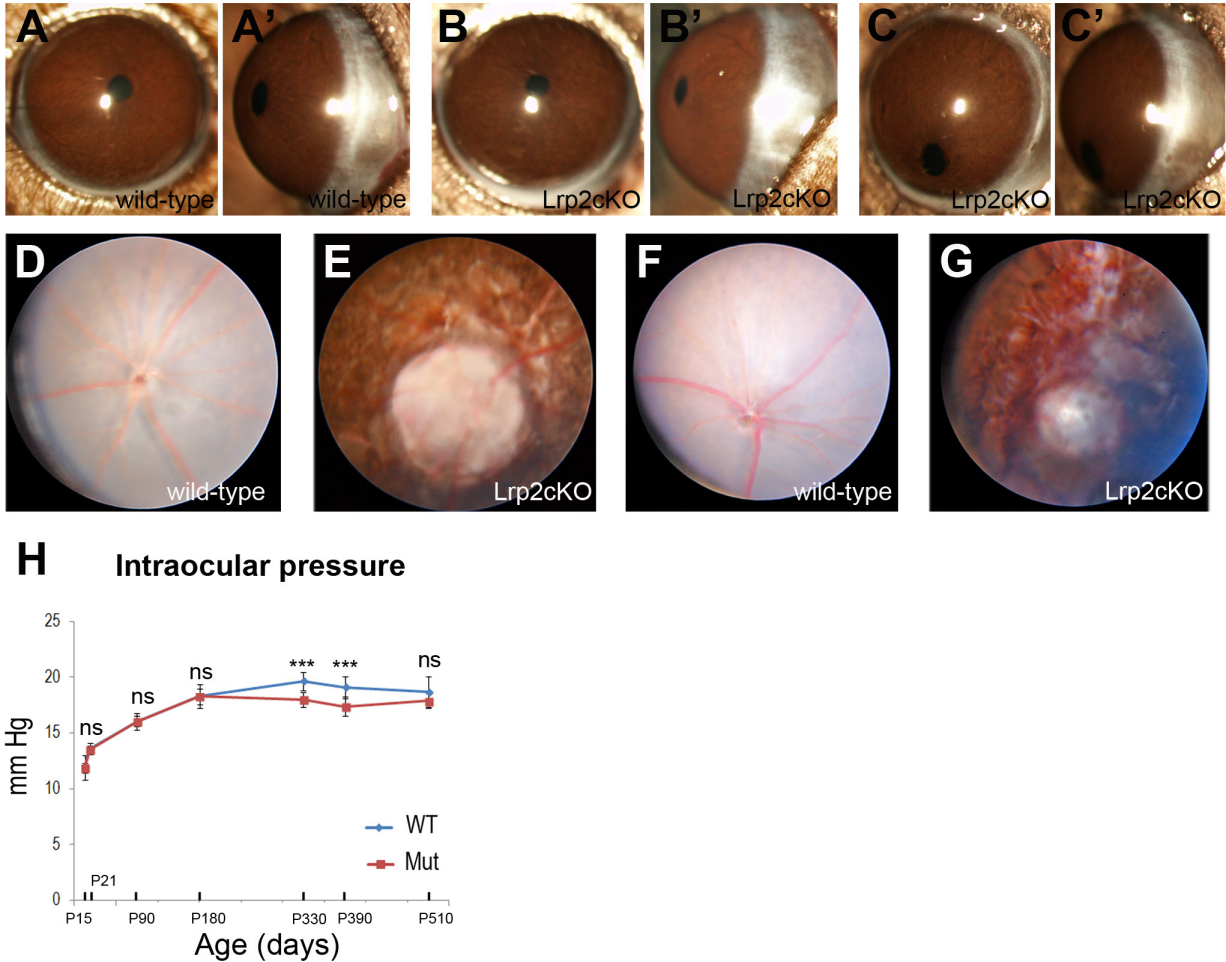
Opthalmological evaluation of control and mutant mice. Similar gross morphology of the anterior segment in control (**A, A’**) and *Lrp2* ^*FoxG1*.*cre-KO*^ mutant littermates at P90 (**B, B’**). In some cases pupillary ectopia was observed in the mutants (**C, C’**). Fundus photographs of control (**D, F**) and mutant eyes (**E, G**) at P60 (**D, E**) and P180 (**F, G**) respectively show chorioretinal atrophy and peripapillary staphyloma. (**H**) Comparisons of intraocular pressure between control and mutant littermates at the indicated postnatal ages. Two-way ANOVA post hoc Tukey test was used, ***P<0.001, ns: not statistically significant, values are mean ± SEM of 10 animals per age and genotype.

Further ophthalmological examination confirmed the MRI data and showed that the anterior segment was generally normal in the mutants (Fig 3A and 3B’) although occasionally pupillary ectopia could be observed (Fig 3C and 3C’). Topical endoscopy fundus imaging at P60 (Fig 3D and 3E) and P180 (Fig 3F and 3G), when the *Lrp2* ^*FoxG1*.*cre-KO*^ mutant eyes were respectively 30% and 40% longer than the control ones revealed that a widespread pigment dispersion was affecting most of the retina. In some cases the visibility of the underlying sclera areas was suggesting a profound chorioretinal atrophy. A peripapillary atrophy of the pigment epithelium consistent with a peripapillary staphyloma, 3–4 disc diameter wide, trait of the so-called myopic retinopathy was also observed [26,27]. Tonometry evaluation showed that the intraocular pressure (IOP) increased rapidly during postnatal development of both control and mutant eyes, and that it was different between the two genotypes (main effect “genotype”: *F*_(1, 126)_ = 39.47, P < 0.0001, interaction: *F*_(6, 126)_ = 11.66, *P* < 0.0001, Fig 3H). Apparently normal values were recorded in the mutants; however the, IOP was significantly lower in *Lrp2*-deficient eyes at P 330 and P390 (***P < 0.001, two-way ANOVA, n = 10 animals per age and genotype, Fig 3H).

It is of interest that normal IOP values can be recorded in highly myopic, including DBS, patients [28]. Together the results show that the lengthening of the ocular axis in *Lrp2* ^*FoxG1*.*cre-KO*^ mutants is primarily due to continuous vitreal chamber enlargement. It is accompanied by myopic chorioretinopathy and normal IOP values

### Early post-natal onset axial elongation in Lrp2^FoxG1.cre-KO^mutant eyes

In highly myopic patients the excessive axial elongation of the eye may be observed early in life and is associated with retinal thinning at both the periphery and posterior pole [8,27,29]. In agreement with this retinal thinning could be seen on the histological and MRI sections of the enlarged mutant eyes from P15 onward (Figs 1F and 2A–2I). To identify the onset of eye enlargement and characterize the mutant retinal morphology we used histological sections of control and mutant eyes at various developmental and post-natal stages.

As shown in Table A-C in S3 Fig retinal morphology appeared normal in the Lrp2^*FoxG1*.*cre-KO*^ mutants between E13.5 and E19.5. Furthermore, the distribution of OTX2, PAX6 and TUJ1, markers which label retinal progenitors, including ganglion cells, and/or RPE cells was not modified in the developing mutant retina between E13.5 and E19.5 (Table A-C in S3 Fig).

**Fig 4:**
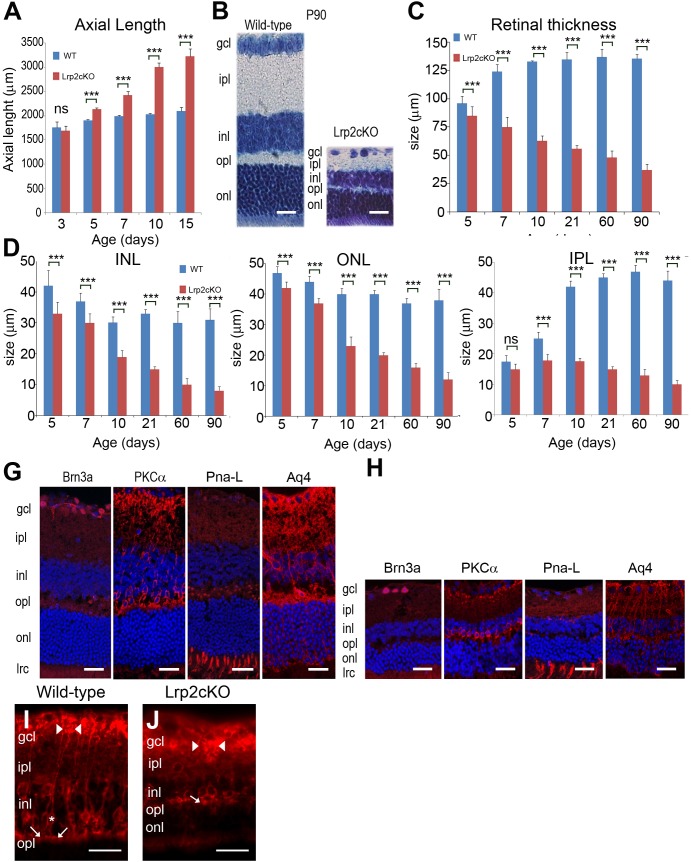
Histological and immunomorphological analysis of post-natal eye growth. Significantly increased axial length is first observed at P5 (**A**). The retinal lamination in P90 *Lrp2* ^*FoxG1*.*cre-KO*^ mutant eyes is normal but all the retinal cell layers appear reduced; Nissl staining (**B**). The continuous reduction of the retinal thickness between P5 and P90 (**C**) is mainly due to the thinning of the inner nuclear (INL), outer nuclear (ONL) and inner plexiform (IPL) cell layers (**D-F**). The distribution of the typical retinal markers Brn3a, PKCalpha, PNA-Lectin (PNA-L) and Aquaporin 4 (Aq4) in the retinal ganglion, bipolar, photoreceptor and Müller cells respectively is similar in control (**G**) and mutant littermates (**H**). The number of PKCalpha, PNA-L and Aq4 positive cells appears decreased in the mutants. (**I**) PKCalpha immunoreactivity in bipolar cells shows a tight cluster of dendrites (arrow) in the OPL, an oval cell body (asterisk) in the INL, and a vertically directed axon, which expands into clusters of terminals (arrowheads) in the proximal portion of the IPL and GCL. (**J**) In the *Lrp2* ^*FoxG1*.*cre-KO*^ mutant the PKCalpha positive bipolar cell has shrunk dendrites at the OPL-INL border (arrow) and a vertically directed axon, which expands abnormally in thick clusters (arrowheads) in the IPL and GCL. Two-way ANOVA post hoc Tukey test was used, **P<0.01, ***P<0.001, values are mean ± SEM of 10 animals per age and genotype. Scale bars: 50 μm in B, G, H; 70 μm in I, J.

Comparative analysis of control and mutant eyes at early post-natal stages did not reveal any differences prior to P3. Between P3 and P15 the AL of the control eyes increased slightly; in *Lrp2*-deficient eyes the AL values were higher and increased significantly between P5 and P15 (two-way ANOVA, main effect “genotype”: *F*_(1, 90)_ = 5019, *P* < 0.0001, interaction: *F*_(4, 90)_ = 746.3, *P* < 0.0001, n = 10 animals per age and genotype, Fig 4A). A significantly higher AL value was first evident in the mutants at P5 (*P* < 0.001, Fig 4A). A histological section of a P5 enlarged mutant eye is shown in Table D in S3 Fig.

Whereas in control eyes the retinal thickness increased sharply between P5 and P21 and then plateaued, in *Lrp2*-deficient eyes it decreased progressively from P5 (Table F and G in S3 Fig) to P90 (Fig 4B) (two-way ANOVA, main effect “genotype”: *F*_(1, 108)_ = 12372, *P* < 0.0001, interaction: *F*_(5, 108)_ = 486.8, *P* < 0.0001, differences between genotypes at each age studied *P* < 0.001, n = 10 animals per age and genotype) (Fig 4C).

We then analyzed the thickness of the inner nuclear (INL), outer nuclear (ONL) and inner plexiform (IPL) retinal layers in control and mutant eyes. In control eyes the thicknesses of the INL, and ONL decreased slightly from P5 to P90; the decrease was more acute in the mutants (two-way ANOVA; INL: main effect “genotype”: *F*_(1, 108)_ = 1142, *P* < 0.0001, interaction: *F*_(5, 108)_ = 46.6, *P* < 0.0001, differences between genotypes at each age studied *P* < 0.001, n = 10 animals per age and genotype, Fig 4D, and ONL: main effect “genotype”: *F*_(1, 108)_ = 5068, *P* < 0.0001, interaction: *F*_(5, 108)_ = 258.5, *P* < 0.0001, differences between genotypes at each age studied *P* < 0.001, n = 10 animals per age and genotype, Fig 4E). Paralleling the increase in connectivity during early postnatal development, the control IPL values increased sharply between P5 and P90 (Fig 4F). In contrast and during the same period the mutant values decreased slightly (two-way ANOVA; main effect: *F*_(1, 108)_ = 12649, *P* < 0.0001, interaction: *F*_(5, 108)_ = 782.6, *P* < 0.0001, n = 10 animals per age and genotype, Fig 4F). A significant reduction of the IPL thickness was observed from P7 onward in the *Lrp2*-deficient retinas (*P* < 0.001, Fig 4F).

To investigate whether retinal thinning was associated with differentiation and/or lamination defects we analyzed the expression of various retinal markers. The differentiated retina cell markers Brn3a, PKCalpha, PNA-Lectin and Aquaporine 4 were similarly distributed in the retinal ganglion, bipolar, photoreceptor and Müller cells (Fig 4G and 4H). It is interesting to note that despite an overall similar distribution of the PKCalpha staining, the mutant bipolar cells had atrophic dendrites and their connections with the retinal ganglion cells appeared thicker (Fig 4J). The above results indicate that despite early onset and excessive thinning the differentiation and lamination of the mutant retina were globally preserved.

### Increased cell-death contributes to excessive retinal thinning in Lrp2^FoxG1.cre-KO^mutant mice

**Fig 5:**
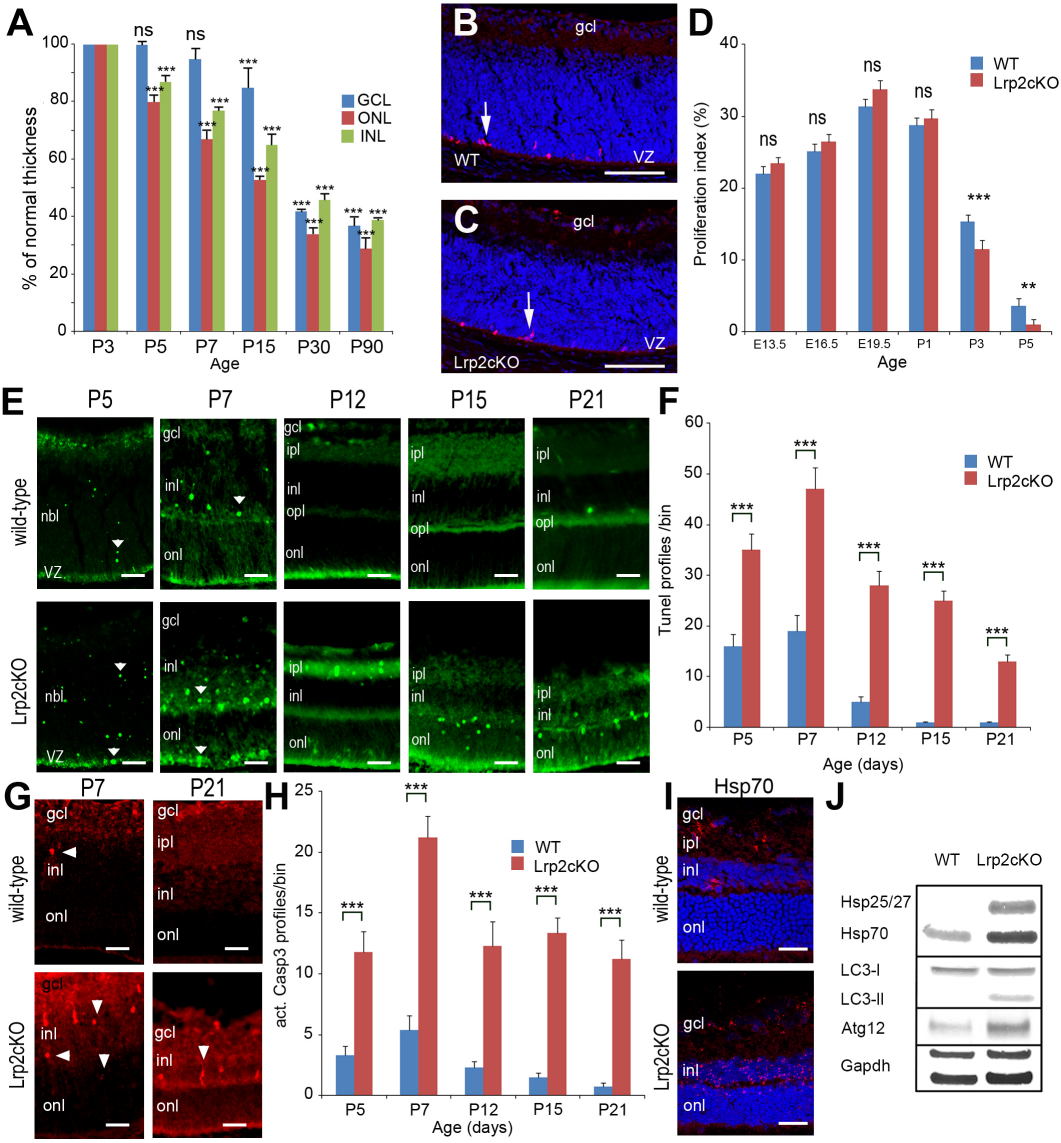
Decreased retinal cell density is associated with increased cell death in *Lrp2* ^*FoxG1*.*cre-KO*^ mutant eyes. Reduced retinal cell density in the ONL, INL and GCL between P5 and P90. Values are expressed in % of normal thickness of the corresponding layers. Comparisons were calculated between P3 as 100% cell density and the other time points in each layer. Two-way ANOVA post hoc Tukey was used, ***P<0.001, ns: non statistically significant, n = 5 animals per age. (**A**). The PH3 + cells are similarly distributed in control (**B**) and mutant retinal layers at P3 (**C**). Similar proliferation indexes in control and mutant retinas between E13.5 and P1, a significant reduction is seen in the mutants at P3 and P5 (**D**). In normal retinas TUNEL + cells are essentially seen in the INL and to a lesser extent in the ONL and GCL layers (**E**, upper panel). In the mutants the TUNEL signal appears stronger in the INL, ONL and IPL (**E**, lower panel). Cell death is significantly increased in the mutants between P5 and P21 (**F**). Distribution of caspase 3+ apoptotic cells in control and mutant retinas at P7 and P21 (**G**). Apoptosis is significantly increased in the mutants (**H**). The expression of the marker of autophagy Hsp70 is particularly strong in the mutant INL (**I**). Western-blot analysis of the indicated autophagic markers; GAPDH is used as an internal loading control (**J**). Two-way ANOVA post hoc Tukey test was used, **P<0.01, ***P<0.001, ns: not statistically significant, values are mean ± SEM of 5 animals per age and genotype; ***p<0.01. Scale bars: 50 μm in B, C, I; 30 μm in E, G.

To investigate whether decreased retinal cell density was contributing to retinal thinning in the mutants we compared the number of cells in control and mutant ganglion (GCL), ONL and INL cell layers using bins of 40,000 square millimeters. From P5 onward progressively decreasing cell density was observed in the mutant ONL and INL whereas in the mutant GCL cell density first decreased at P15 (two-way ANOVA; main effect “age”: *F*_(5, 72)_ = 3429, *P* < 0.0001; main effect “genotype”: *F*_(2, 72)_ = 569.8, *P* < 0.0001, interaction: *F*_(10, 72)_ = 78.2, *P* < 0.0001, n = 5 animals per age and genotype, Fig 5A). Decreased cell density is generally due to insufficient cell proliferation and/or increased cell death and may also reflect the continuous stretching of the mutant retina. We therefore analyzed the expression of cell proliferation and death markers at various developmental and post-natal stages. The M-phase cell cycle marker phospho-histone H3 (PH3) was normally distributed in the mutant retinal progenitor cells between E13.5 and P1 (Table A in S4 Fig), as well as at P3 (Fig 5B and 5C). The pattern of the proliferation index was globally similar in both genotypes between E13.5 and P1 (two-way ANOVA, main effect “genotype”: *F*_(1, 48)_ = 5.667, *P* = 0.0213, main effect “age”: *F*_(5, 48)_ = 1763, *P* < 0.0001, interaction: *F*_(5, 48)_ = 6.385, *P* = 0.0001, n = 5 animals per age and genotype, Fig 5D). A significant reduction of the proliferation index was seen at P3 and P5 in the *Lrp2*-deficient eyes (P3, *P* < 0.001, and P5, *P* < 0.01, Fig 5D).

We used TUNEL-labeling to identify cell death of retinal neurons before birth and between P5 and P21 when programmed cell death peaks in mouse retina [30]. In control retinas TUNEL positive cells were observed between E13.5 and P1 (Table B in S4 Fig). At P5 TUNEL-positive cells were mainly seen in the neuroblastic layer (NBL) of the developing control retina (Fig 5El). At P7, the NBL had differentiated into the ONL and INL where most of the TUNEL-positive cells were localized (Fig 5E). From P12 onward, programmed cell death had mostly subsided and only a few TUNEL-positive cells were observed in the control retinas (Fig 5E). Compared with the controls the distribution of the TUNEL positive cells was not modified in the mutants, prior to P1 (Table B in S4 Fig). The number of TUNEL-positive cells appeared however higher in the mutant retinas between P5 and P21, especially in the INL, ONL and inner plexiform cell layers (Fig 5E). Accordingly, the quantitative analysis clearly showed a significantly higher number of TUNEL-positive cells in the mutant retinas between P5 and P21 (two-way ANOVA followed by post hoc Tukey test, main effect “genotype”: *F*_(1, 40)_ = 3681, *P* < 0.0001, interaction: *F*_(4, 40)_ = 57.17, *P* < 0.0001, each age *P* < 0.001, n = 5 animals per age and genotype, Fig 5F). Activated caspase 3 is a pro-apoptotic mediator, expressed during retinal development. In control retinas the activation of caspase 3 peaked in the INL at P7 whereas at P21 only a few caspase 3-profiles were observed throughout the retina (Figs 5G and 4H). In agreement with increased TUNEL staining, the mutant retinas also showed a significantly higher number of caspase 3-profiles between P5 and P21 (two-way ANOVA, main effect: *F*_(1, 40)_ = 1708, *P* < 0.0001, interaction: *F*_(4, 40)_ = 18.86, *P* < 0.0001, each age *P*<0.001, n = 5 animals per age and genotype, Fig 5G and 5H)

We then compared the expression of stress-related markers in control and mutant eyes. At P21 the expression of the heat shock protein Hsp70 [31] was increased in the mutant INL (Fig 5I). Because stress-related proteins are also actors of the chaperone-mediated autophagy we hypothesized that this pathway might contribute to cell death in the mutants. This hypothesis was supported by the high expression of markers widely used to monitor autophagic activity including Hsp25/27, the autophagosome marker microtubule-associated protein light chain LC3B-II and the autophagy-related gene Atg12 exclusively in the mutant retina (Fig 5J). Finally GFAP, another stress-related marker that labels astrocytes was strongly expressed in the mutant ganglion cell layer (Table A and B in S5 Fig) further suggesting that retinal thinning and retinal cell death, at least partly due to increased stress were associated events in the Lrp2^*FoxG1*.*cre-KO*^ eye.

Cell death was only occasionally observed in control retinas after P21. In the *Lrp2* ^*FoxG1*.*cre-KO*^ mutant retinas however, cell death appeared to increase especially in the mutant ganglion cell layer and consequently led to a reduction of the number of axons in the mutant optic nerve observed at P150 (two-tailed, unpaired *t* test p*** = 1.07E-6<0.001, n = 4 animals per age and genotype, Table C in S5 Fig). It is interesting to note that despite the reduction of the number of axons in the mutant optic nerve at P150 the optic nerve diameter was slightly increased in the mutants (two-tailed, unpaired *t* test p* = 0.022<0.05, n = 4 animals per age and genotype, Table D in S5 Fig).

### Scleral modifications in Lrp2^FoxG1.cre-KO^mutant mice

The sclera, the outer coat of the eye, is a collagen and proteoglycan containing connective tissue with flattened fibroblasts embedded in it. Thinning of the sclera, in particular at the posterior pole of the eye and the ensuing posterior staphyloma, an outward protrusion of all posterior layers, are typical features of myopic maculopathy [9,32].

**Fig 6:**
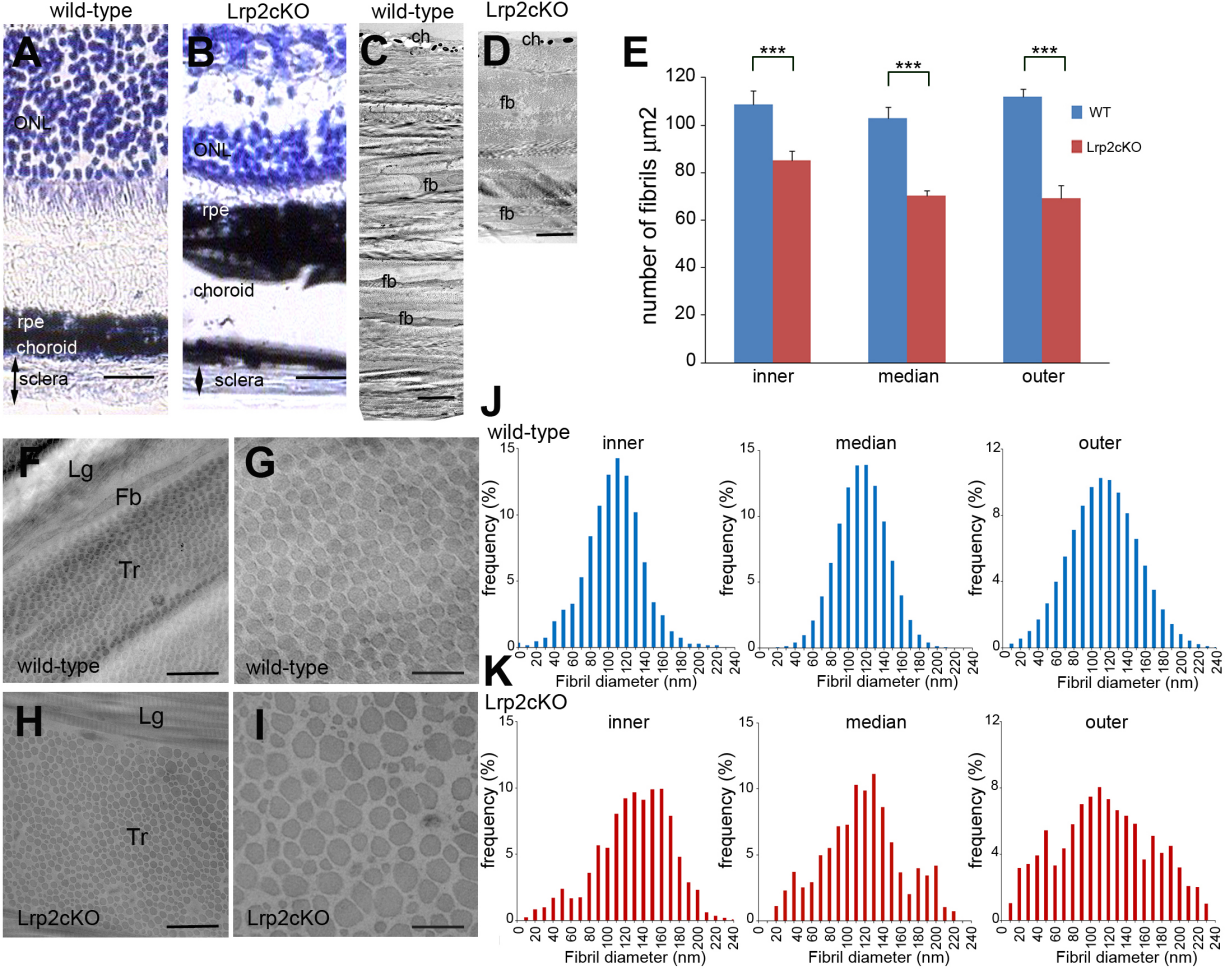
Scleral modifications in *Lrp2* ^*FoxG1*.*cre-KO*^ mutant eyes. Nissl staining of retinal sections in control (**A**) and mutant (**B**) eyes shows reduced scleral thickness at P90. The choroid and occasionally the RPE appear thicker at the posterior pole of the mutant eyes. Transmission electron micrographs of the posterior sclera wall (**C, D**) shows that the collagen fibrils form well-organized lamellae in the control sclera (**C**); in the mutant fibril-poor areas and impaired packing are evident (**D**). Contrary to the control sclera the collagen fibril density is lower in all layers of the mutant sclera (**E**). Transmission electron micrographs showed fibril collagen organization within a lamella of the posterior sclera (**F, H**) and in localized areas (**G, I**) of control (**F, G**) and mutant (**H, I**). Fibrils were morphologically abnormal with irregular contours and heterogeneous diameters in the mutants. Measurements of cross-sectional diameters of fibrils from the inner, middle and outer posterior sclera in control (**J**) and mutant eyes (**K**). The mean fibril diameter distribution is modified in the mutants in all three layers; the frequency of very small as well large diameter fibrils is increased. (ch) choroid, (fb) fibroblast, (Lg) longitudinal and (Tr) transversal orientation of the cross-sectioned fibrils. Two-tailed unpaired *t* test was used. ***P<0.001, Values are mean ± SEM of 3 animals per genotype; see methods. Scale bars: 50 μm in A, B; 3.5 μm in C, D; 1.2 μm in F, H; 300 nm in G, I.

At the posterior pole of control P90 eyes the mean scleral thickness was around 35μm (Fig 6A). At the same age the scleral thickness of the *Lrp2* ^*FoxG1*.*cre-KO*^mutant eyes appeared reduced (Fig 6B). Comparison of the mean scleral thickness at P90 and P180 showed that at these ages the mutant sclera was thinner than the control one by 33% and 50% respectively (two-tailed, unpaired *t* test. P90, p*** = 0.000255<0.001; P180, p*** = 3.99E-4<0.001, n = 3 animals per age and genotype, Table A in S6 Fig).

Ultrastructural analysis revealed that while in the control sclera collagen fibrils were organized into well-defined lamellae (Fig 6C) the mutant collagen fibrils formed fewer lamellae at P90 and P180 (two-tailed, unpaired *t* test. P90, p** = 0.0018<0.01; P180, p*** = 0.00027<0.001, n = 3 animals per age and genotype, Table B in S6 Fig) which appeared disorganized (Fig 6D). Further analysis of P90 control and mutant eyes showed that within the mutant lamellae the organization of the collagen fibrils was also perturbed; interweaving fibrils, readily observed in the controls, were only occasionally seen throughout the mutant sclera (Table C–J in S6 Fig). The mutant fibrils seemed to course parallel to each other and large areas devoid of fibrils were often found between the lamellae of the inner, middle and outer sclera (Table C–H in S6 Fig). Moreover the mutant fibrils appeared abnormally drawn compared with the controls (Table I and J in S6 Fig). Quantification of the scleral fibrils per square millimeter showed that the fibril density was significantly lower in the mutants (two-tailed, unpaired *t* test, inner: ***P<0.001, t = 20.63, df = 22; median: ***P<0.001, t = 24.97, df = 22; outer: ***P<0.001, t = 41.9, df = 22, n = 3 animals per genotype, Fig 6E) and that, in contrast with the controls, it was lower in the outer sclera (Fig 6E).

The morphology of the mutant fibrils was very heterogeneous even within a single region of the sclera and their contour was irregular with rectangular- rather than oval-shaped fibrils readily found throughout the mutant sclera (Fig 6F–6I). In control eyes the majority of collagen fibrils from the outer, middle and inner layers of the posterior sclera had a mean cross-sectional diameter of ~115 nm (Fig 6J). Compared with the controls the mean cross-sectional diameter of the mutant fibrils was drastically modified in the three scleral layers (Fig 6K). It was of~135 nm in the inner and middle scleral layers and ~120 nm in the outer layer. The frequency however of fibers with both smaller (<60 nm) and wider (>180 nm) mean cross-sectional diameters was increased in the mutants (Fig 6K). The above results indicate that the posterior mutant sclera undergoes important structural modifications reminiscent of human HM [33] and consistent with the development of posterior scleral staphyloma.

## Discussion

In the present study we show that Lrp2 expressed in the anterior neuroepithelium and its derivatives the neural retina, ciliary and retinal pigment epithelium is required for normal eye growth. *Lrp2* deficient eyes are abnormally enlarged essentially along the anterior-posterior axis, a feature typical of high myopia. The continuous eye elongation is accompanied by posterior segment anomalies including chorio-retinal atrophy and posterior staphyloma, features of the myopic retinopathy or maculopathy [1,9,27].

LRP2 is a large plasma membrane protein generally expressed at the apical pole of absorptive epithelia. Prominent sites of expression include the renal proximal tubules, the ependymal cells of the brain and the developing neuroepithelium [34,35]. In the kidney LRP2 acts as a clearance receptor for filtered plasma proteins including steroid hormones and carrier proteins for vitamin D metabolites and retinoids, preventing uncontrolled loss of these metabolites and regulating systemic vitamin homeostasis. Mice carrying a targeted disruption of the *Lrp2* gene suffer from forebrain malformations, lack of corpus callosum and impaired eye development [16,22]. Most of them die within minutes after birth with only a small percentage surviving to adulthood [36]. Conditional inactivation of *Lrp2* using a floxed *Lrp2* allele [19] and the *MORE*-Cre active in the epiblast but not the extra-embryonic tissues results in an identical phenotype confirming the significance of embryonic LRP2 in brain and eye development [16]. In the *Lrp2*-deficient adult mice the brain and eye defects are associated with low molecular weight proteinuria and vitamin deficiency [17,36]. Remarkably, the nonsense mutations in the zebrafish *lrp2 bugeye* mutant cause eye enlargement and overt renal reabsorption deficits [13,37] phenotypes shared by patients suffering from DBS [11]. These studies clearly suggest that LRP2 functions are evolutionary conserved but do not provide any information on the tissue autonomy of the ocular LRP2 function.

The LRP2 expressing tissues involved in ocular structure formation are the neuroepithelium that forms the retina, the RPE and the ciliary body epithelium and the neural crest that gives rise to the central part of cornea [38]. LRP2 is also strongly expressed in the optic nerve astrocytes [24]. To preserve the renal function of LRP2 and identify the LRP2 expressing tissue required for normal eye growth we selectively inactivated *Lrp2* in the anterior neuroepithelium, the neural crest or the astrocytes. Whereas *Lrp2* inactivation in the neural crest, *via Wnt1-Cre*, or the astrocytes *via GFAP-Cre* did not alter eye morphogenesis, *FoxG1*-mediated ablation of *Lrp2* dramatically modified post-natal eye development identifying *Lrp2* expressed in the anterior neuroepithelium and its derivatives as an essential component of eye morphogenesis.

*FoxG1*-mediated gene ablation is first efficient around E9.0 [20], stage at which the single eye field is already separated into two, forming the optic vesicle and later, the optic cup. The formation of the optic cup as well as further differentiation of the neuroretina and RPE depends among others on BMP and SHH signaling, pathways known to be modulated by LRP2 [16,22]. Optic cup formation, subsequent retinal differentiation followed by the expression of OTX2, PAX6 and TUJ1, as well as rates of retinal cell proliferation and death appear normal between E13.5 and P5 suggesting that these signaling pathways are unlikely to be perturbed in the *Lrp2*-deficient eyes. Further retinal differentiation is overall preserved as suggested by the normal distribution of Brn3a, PKCalpha, PNA-Lectin and Aquaporin 4.

The excessive lengthening of the *Lrp*2^*FoxG1*.*cre-KO*^ mutant ocular axis begins around P5 and is almost exclusively due to the elongation of the vitreal chamber. Indeed the overall growth and morphology of the anterior segment are only marginally modified in the mutants. With the exception of the slight, albeit significant reduction of the lens thickness observed around P330, the CRC and ACD values as well the size of the pupil are not modified in the *Lrp2*-deficient eyes. Furthermore we do not find any sign of cataract and the occasionally observed pupillary ectopia may be secondary to a detachment of the ciliary body due to the excessive elongation-associated traction.

In addition to the enlarged vitreal chamber the modifications of the *Lrp2*-deficient posterior segment include retinal thinning and chorioretinal atrophy. Retinal thinning is not simply due to the continuous eye growth but also to a slightly reduced cell proliferation and increased retinal cell death. Between P5 and P21 cell death is particularly important in the photoreceptor and bipolar cell layers. After this age increased cell death in the retinal ganglion cell layer eventually leads to the decrease of the RGC axon number. Remarkably the expression of several autophagic markers is abnormally strong suggesting that the auto regulatory capacities of the *Lrp2*-deficient retinas may be affected. It is possible that the retinal modifications provide signals contributing to the continuous eye growth but it is not clear how these signals may be triggered from P5 onward. Because the eye-lids are still closed at this age and the retina does not process visual information, it is unlikely that the onset of abnormal eye-growth is visually driven in the mutants. It is rather due to signals produced by the *Lrp2* deficient tissues. The ciliary epithelium and RPE are the only sites that express LRP2 throughout life. In view of the implication of LRP2 in protein endocytosis *in vivo* [17] it is possible that LRP2 mediates the selective uptake of macromolecules and delivery of vitamins or other nutrients to the ocular structures. Whether the impairment of this function provides signals for abnormal eye growth remains to be established.

Scleral modifications including thinning and formation of posterior staphyloma are first evident around P21 suggesting that they are the consequence rather than the cause of the excessive axial elongation in the *Lrp*2^*FoxG1*.*cre-KO*^ mutants. Although the study of the biomechanical properties of the mutant sclera is beyond the scope of the present work one would anticipate that scleral thinning, impaired collagen fibril number and morphology may increase scleral extensibility and thus favor the Lrp2^*FoxG1*.*cre-KO*^ mutant eye enlargement and formation of peripapillary staphyloma. Increased scleral extensibility may also at least partly explain the rather normal intraocular pressure observed in the Lrp2^*FoxG1*.*cre-KO*^ mutant eyes as well as in highly myopic patients [28].

In sum the present work shows that LRP2 expressed in the developing ocular tissues is required for normal eye growth and that the *Lrp2* ^*FoxG1*.*cre-KO*^ mutants share many characteristics of congenital HM. Because the pathological significance of HM is not due to ametropia but rather the development and extent of the associated degenerative changes we believe that the Lrp2^*FoxG1*.*cre-KO*^ mutants provide a unique tool to study the onset and age-related evolution of high myopia.

## Methods

### Ethics statement

Animal procedures were conducted in strict compliance with approved institutional protocols (INSERM and comité d’éthique en experimentation animale Charles Darwin N°5, permit number 01519.01) and in accordance with the provisions for animal care and use described in the European Communities council directive of 22 September 2010 (2010/63/EU). Deep anesthesia for terminal procedures (perfusion) was provided with a ketamine/xylazine cocktail (80mg/10mg/kg).

### Animals

*Foxg1*.Cre (129.Cg-*Foxg1* ^*tm1(cre)Skm*^/J)[20], *Wnt1*-Cre (Tg(Wnt1-cre)11Rth/MileJ)[39] mice were purchased from the Jackson Laboratory (Bar Harbor, Maine). h*GFAP*-Cre [40] and *Lrp2* ^*Lox/Lox*^ [19] mice have been described elsewhere. Gestation (E0.0) was considered to have begun at midnight before the morning when a copulation plug was found. For each experimental analysis 3–10 mice per age and genotype were used; the exact number appears in the results and figure legends sections.

### Immunohistochemistry-Immunofluorescence

Whole embryos were immersed in a solution of 4% paraformaldehyde in PBS for 1 hour at 4°C on a rocking platform. Embryos were frozen and cut into sections 10 μm thick. Pups and adults were anesthetized and perfused transcardially with 4% PFA in 0.12 m phosphate buffer, pH 7.4. After perfusion, eyes were removed from the skull and postfixed overnight in fresh fixative. Serial frozen sections were processed for immunocytochemistry using the antibodies listed in S1 Table Lectin PNA conjugates (1/50; L-21409, Molecular Probes, OR 97402) were also used. Alexa 488- or 594-conjugated antibodies (1:200, Invitrogen) were used for secondary detection. Nuclear staining was achieved in Hoechst 33342. Fluorescent images were obtained using an Olympus confocal microscope (FV-1200-IX83).

### Morphometric analysis

For all quantification, slides were coded and counts were performed with the examiner blind to the age and genotype. Coronal sections of wild-type, and *Lrp2* ^*FoxG1*.*cre-KO*^ mice were Nissl stained and analyzed using a 40× objective and a millimetric eyepiece. For each specimen, the retinal thickness and the number of cells were estimated on the two eyes in ten sections spaced by 40 μm. The number of Nissl profiles was counted in an area of 40,000 μm^2^ in the GCL, INL, and ONL. The count was performed in a region excluding the ciliary margin and the optic nerve head. For optic nerve studies sections of P45 and P150 wild-type and mutant mice were processed for immunohistochemistry. Fluorescent area and optic nerve diameter were measured by Fiji-ImageJ software.

### Terminal deoxynucleotidyl transferase-mediated biotinylated UTP nick end labeling staining

Pups and adults were anesthetized, and their eyes were immediately removed and frozen in isopentane. To visualize nuclei with DNA cleavage, serial sections (20 μm) of the eye were cut on a cryostat, and residues of fluorescein-labeled nucleotides were catalytically added to DNA fragments by terminal deoxy-nucleotidyl-transferase (TdT). Briefly, sections were fixed in fresh 4% PFA/PBS at room temperature for 15 min, washed in PBS three times for 5 min, equilibrated at room temperature for 10 min, and incubated with nucleotide mix and TdT (ApoAlert DNA fragmentation kit; Clontech, Mountain View, CA) at 37°C for 1 h. Tailing reaction was stopped by incubating sections in 2× SSC at room temperature for 15 min. The number of apoptotic profiles was counted in an area of 40,000 μm^2^ in the retina. The count was performed in a region excluding the ciliary margin and the optic nerve head.

### High resolution MRI

Mice were anesthetized with a mixture of isoflurane (5% for induction, 1–2% for maintenance) air (1 L/min) and dioxygene (0.2 L/min). 100 μl of MRI contrast agent (Gd-DOTA, Dotarem, Guerbet, France) were injected intra-peritonealy on each mouse prior to MRI scans. In vivo MRI scans were performed on an 11.7T spectrometer (117/16 USR Biospec, Bruker Biospin, Ettlingen, Germany) equipped with a BGA9-S gradient insert (740 mT/m, rise time 100μs) and interfaced with a console running Paravision 5.1. An MRI Cryoprobe dedicated to mouse imaging was used both for emission and reception of the NMR signal. A fast 2D-gradient echo sequence (FLASH) was used to position slices bisecting the optic nerve of the mouse eye (TR/TE = 567.1/3.7 ms, Nex = 1, Mtx = 384*256, field-of-view = 2.30*1.54 cm yielding an in-plane resolution of 60 μm with a slice thickness of 200 μm on 40 slices, Acquisition Time = 2min and 25s). Then, a two-dimensional (2D) T1-weighted spin-echo sequence was acquired (TR/TE = 1500/10.5 ms, Nex = 2, Mtx = 512*256, field-of-view = 2.56*1.28 yielding an in- plane resolution of 50 μm with a slice thickness of 120 μm on 48 slices, Acquisition Time = 12min and 48s) to distinguish vitreous from aqueous humour and allow detection of any leakage from one chamber to another. Finally, a high resolution 2D T2-weighted spin-echo sequence (TR/TE = 4000/25.8 ms, Nex = 1, Mtx = 1024*512, field-of-view = 3.17*1.58 cm yielding an in-plane resolution of 31 μm with a slice thickness of 120 μm on 48 slices, Acquisition Time = 34min and 8s) was used to obtain a variety of ocular parameters. We measured axial length (AL), equatorial diameter (ED), anterior chamber depth (ACD), vitreous chamber depth (VCD), anterior chamber width (ACW), and lens thickness (LT) as defined by Tkatchenko et al. 2010 [25]. The ACD and ACW were used to calculate the corneal radius of curvature (CRC) as CRC = (ACD/2) + (ACW^2^/(8 X ACD)).

### Topical endoscopy fundus imaging

Topical endoscopy fundus imaging (TEFI) was performed [41] with an endoscope with a 5-cm long otoscope with a 3-mm outer diameter (1218AA; Karl Storz, Tuttlingen, Germany) with step index lenses and an angle of view of 0°, a field of view of 80° in air, and a crescent-shaped illuminating tip. A reflex digital camera with a 6.1-million pixel charge-coupled device (CCD) image sensor (D50 with Nikkor AF 85 /F1.8 D objective; Nikon, Tokyo, Japan) was connected to the endoscope through an adapter containing a +5 lens (approximate value; the distance from the tip to the cornea appeared to have more influence on focus than did the lens power). The preferred settings of the camera are as follows: image format, raw; focus, manual; operating mode, A (priority to opening); diaphragm, 1/1.8; white balance, automatic. The light source was a xenon lamp (reference 201315–20; Karl Storz) connected through a flexible optic fiber to the endoscope.

### Anterior Segment and Tonometry Evaluation

Anterior chamber phenotypes were assessed with a slit lamp (SL-D7, Topcon, Oakland, NJ) and photo-documented with a digital video camera (DV-3, Topcon). All images were taken using identical camera settings and prepared by processing with identical image software. All ocular examinations were performed on conscious mice.

### Intraocular Pressure (IOP) Measurement

IOP was measured using the TonoLab rebound tonometer for rodents (Tiolat i-care, Finland) according to the manufacturer’s recommendations. All IOP measurements were performed between 10 AM and noon in conscious condition. Mice were gently restrained first by hand and placed on a soft towel bed on the desk and usually appeared calm and comfortable. These data were confirmed to be reproducible by three additional different independent studies (n = 20).

### Transmission Electron Microscopy

Adult mice were anesthetized and perfused transcardially with 2% paraformaldehyde 2.5%glutaraldehyde in 0.1M PBS (pH 7.4). Retinas were sliced in 200-μm-thick sections, postfixed 2 h in 1% osmium tetroxide, dehydrated in alcohol, cleared in acetone, and embedded in Epon. For light microscopy, transverse serial sections (1 μm) were cut, heat dried, and stained with toluidine blue. Ultrathin sections were cut and stained with lead citrate and examined with a Philips (Aachen, Germany) CM100 electron microscope.

### Fibril diameter analyses

For each genotype, three different animals were analyzed. Only the posterior sclera was analyzed in detail. For the posterior sclera, micrographs (12 per group) from nonoverlapping regions of the central portion of sclera wall were taken at X31,680. The diameter of 85 to 300 fibrils was measured from a single region of a photographic negative. For fibrils that contained uneven contours, the minimum diameter was included in the analysis. Micrographs were randomly chosen in a masked manner from the different groups and digitized, and diameters were measured in an image analysis system (FIJI).

### SDS-PAGE and Western blot analysis

SDS-PAGE and immunoblotting were performed as previously described [23]. Briefly 15 mg of retina were separated by SDS-PAGE and transferred to nitrocellulose membranes. After blocking the membranes were incubated with the following primary antibodies: mouse anti-HSP25/27 (1/5000; SMC-163C/D, StressMarq), and anti-HSP70 (1/5000; SMC-114C/D, StressMarq), rabbit anti-LC3B (1/1000; #2775, Cell Signaling, MA 01915) and anti-Atg12 (1/1000; #2011, Cell Signaling). Mouse anti-GAPDH (1/500; SC-166545, Santa Cruz) was used as an internal control. Immunoblots were washed in TBS–0.02% Tween 20 (TBST), incubated for 1 h with the appropriate horseradish peroxidase-coupled IgG (diluted 1:5000), washed in TBS–0.02% Tween 20, revealed using Super Signal (Pierce, Rockford, IL), and exposed to Hyperfilm ECL (Amersham Biosciences). Silver staining using the Biorad Silver Stain Plus kit was performed according to the manufacturer’s instructions.

### Data analyses and statistics

All data are expressed as the means ± s.e.m. To determine significance in our comparisons of biometric and histologic measurements, we used a commercial software (GraphPad Prism 6, San Diego, CA) for two-way ANOVA, with one factor, to determine the main effects (genotype and postnatal day) and their interaction. Differences, when significant (p<0.05) between genotypes at specific postnatal days were analyzed by the Tukey post hoc test and added in the graph. When only two groups were compared (Fig 6E, S5C and S5D, S6A and S6B Figs), two-tailed unpaired Student's *t* test was used.

## Supporting Information

S1 Fig Normal retinal morphology in Lrp2^*Wnt1*.*cre-Ko*^ and Lrp2^*GFAP-cre-KO*^ mutants. Nissl staining of retinal layers. Scale bars: 50 μm in A, B.
(TIF)

S2 Fig MRI and histological analysis of control and *Lrp2* ^FoxG1.cre-KO^ mutant eyes. Parameters extracted from high resolution MRI images (**A-C**). Asphericity coefficient was calculated as an AL/ED ratio; circumference and area are increased in the mutants. Two-way ANOVA post hoc Tukey test was used; ***P<0.001, values are mean ± SEM of 4 animals per age and genotype.
(TIF)

S3 Fig Retinal development in control and *Lrp2* ^FoxG1.cre-KO^ mutant eyes. Retinal cryosections of control and mutant eyes between E13.5 and E19.5 show similar distribution of OTX2 (**A**), PAX6 (**B**) and TUJ-1 (**C**) in the developing retina. Nissl staining on sagittal cryosections of control and mutant eyes at P5 showing increased AL (black double-headed arrow) and equatorial diameter (red double-headed arrow) in the mutants (**D, E**). Retinal thickness is reduced in the mutants (**F**, **G**). Scale bars: 25 μm A-C; 300 μm in D, E, 90 μm in F, G.
(TIF)

S4 Fig Cell proliferation and death in the developing retina of control and *Lrp2* ^FoxG1.cre-KO^ mutant eyes. The cell proliferation PH3 immunostaining (green) is similarly distributed in the ventricular zone (vz) of control and mutant eyes at the ages indicated (**A**). TUNEL + cells (white arrows) are occasionally found in control and mutant retinas at the ages indicated (**B**). Scale bars: 25 μm in A, B.
(TIF)

S5 Fig Increased GFAP retinal staining and reduction of the number of axons in the *Lrp2* ^FoxG1.cre-KO^ mutant eyes. GFAP staining on retinal cryosections of control and mutant retinas at P10 (**A**) and P90 (**B**). The signal is increased in the astrocytes (arrows) of the mutants. Neurofilament Heavy Chain (NFH) staining is used to count the optic nerve axons at the ages indicated (**C**). Despite the significant reduction of NFH+ fibers (55%) observed at P150 in the mutant optic nerve, the diameter of the mutant optic nerve is slightly higher than that of the control (**D**). Comparisons are made between the two groups, age matched controls and *Lrp2* ^*FoxG1*.*cre-KO*^ by two-tailed, unpaired *t* test. C: P45, p = 0.099; P150, p*** = 1.07E-6<0.001. D: P45, p = 0.23; P150, p* = 0.022<0.05. Values are mean ± SEM of 4 animals per age and genotype. Scale bars: 50 μm in A; 30 μm in B.
(TIF)

S6 Fig Scleral modifications in the the *Lrp2* ^FoxG1.cre-KO^ mutant eyes. Reduced posterior sclera thickness (**A**) is associated with a reduction in the number of lamellae across the entire mutant sclera (**B**) at the ages indicated. Transmission electron microscopy reveals collagen architecture in the inner, middle and outer layers of the posterior sclera in control (**C, D, E, I**) and mutant (**F, G, H, J**) eyes at P90. Decreased interweaving, abnormal collagen packing of the mutant fibrils and fibril-free spaces, asterisks in (**F**, **G**), are seen throughout the mutant retina. Comparisons are made between the two groups, age matched controls and *Lrp2* ^*FoxG1*.*cre-KO*^ by two-tailed, unpaired *t* test. A: P90, p*** = 0.000255<0.001; P180, p*** = 3.99E-4<0.001. B: P90, p** = 0.0018<0.01; P180, p*** = 0.00027<0.001. Values are mean ± SEM of 3 animals per age and genotype. Scale bars: 1 μm in C-H; 600 nm in I, J.
(TIF)

S1 Table List of antibodies used in histology. (DOCX)
