# Supplementary figures and images for: Foxg1-Cre Mediated Lrp2 Inactivation in the Developing Mouse Neural Retina, Ciliary and Retinal Pigment Epithelia Models Congenital High Myopia
Source: PLoS One. 2015 Jun 24;10(6):e0129518. doi: 10.1371/journal.pone.0129518 (PMC4480972; doi:10.1371/journal.pone.0129518)

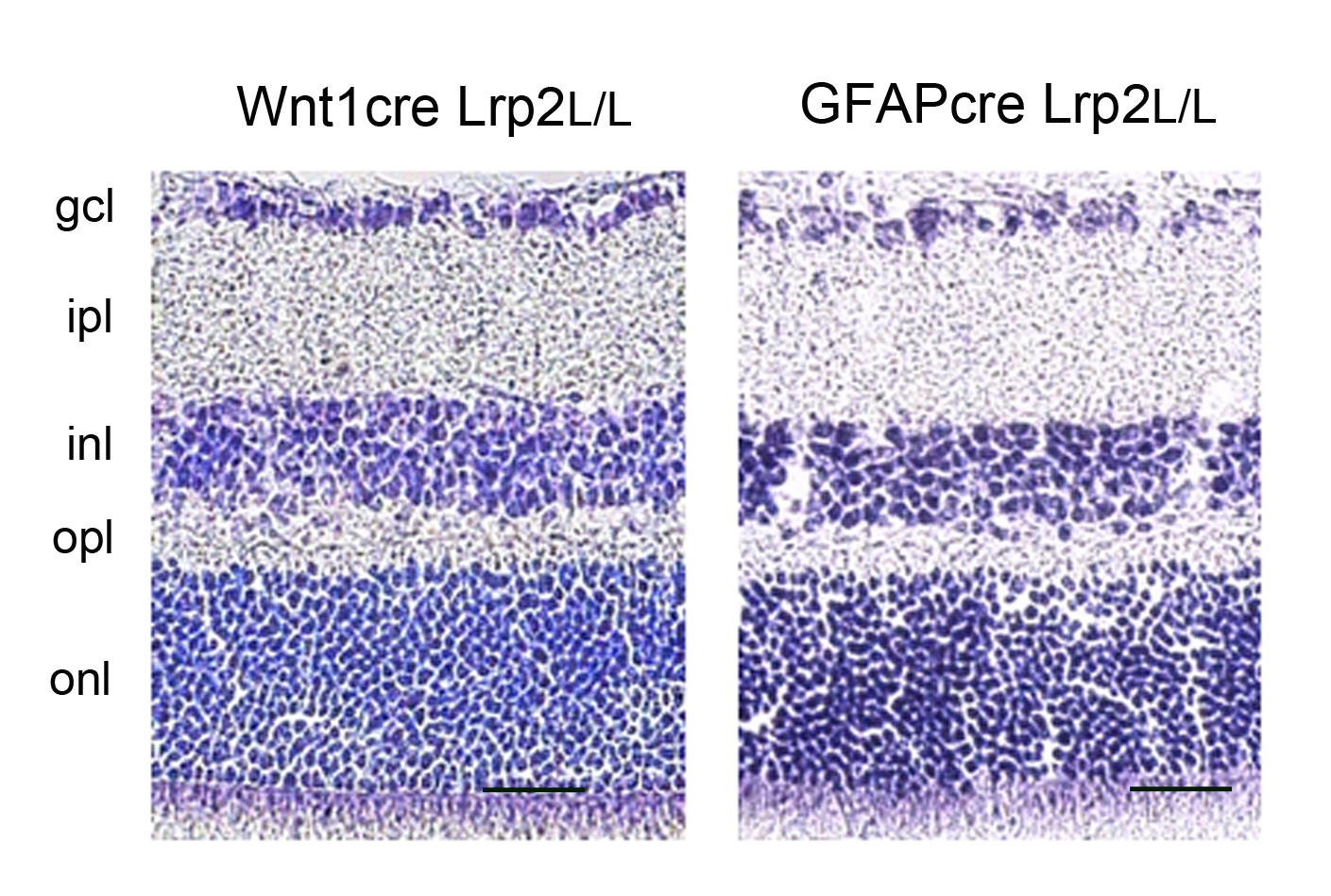

Supplement: S1 Fig — Nissl staining of retinal layers. Scale bars: 50 μm in A, B. (TIF) [file pone.0129518.s001.tif]

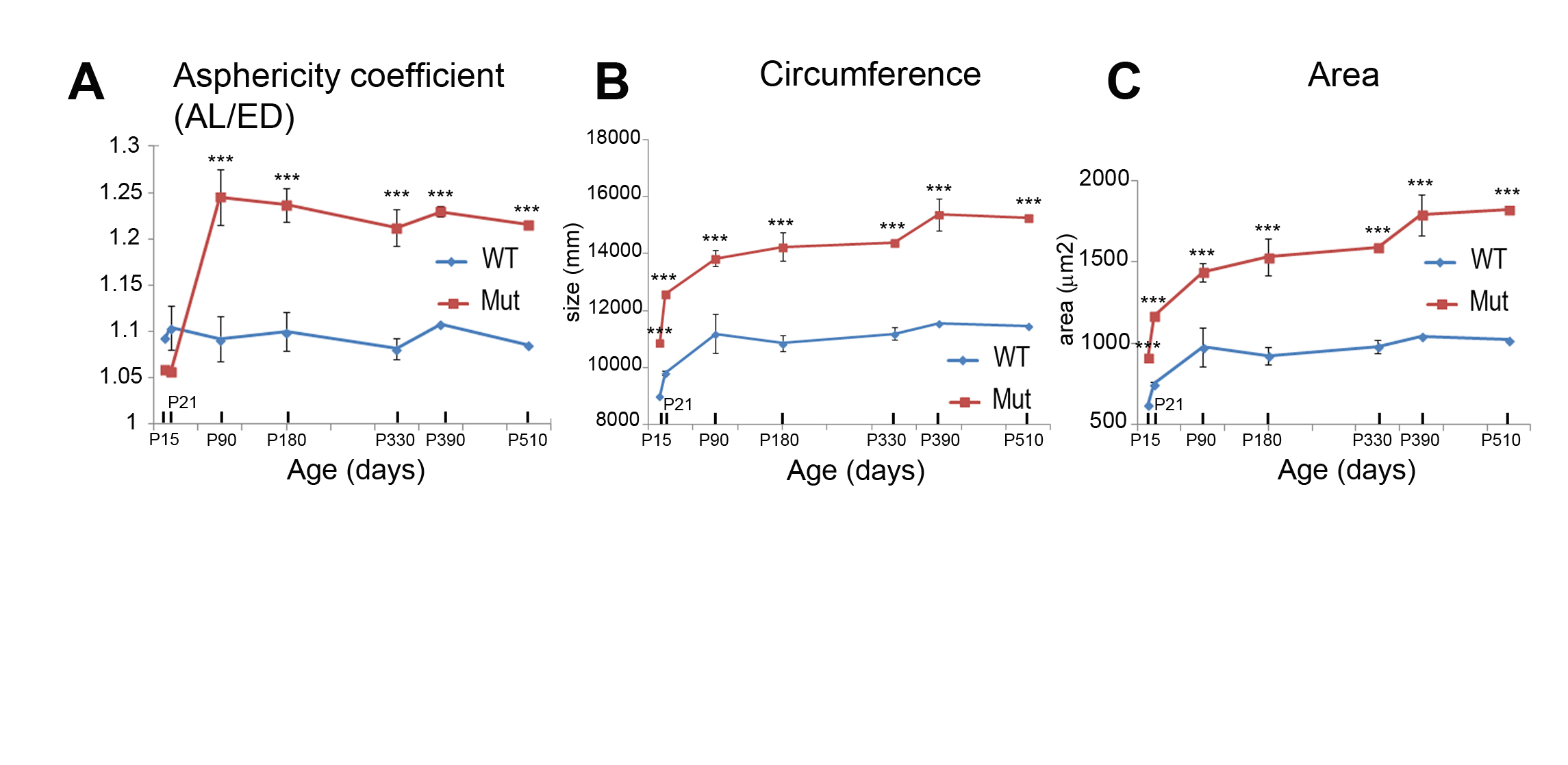

Supplement: S2 Fig — Parameters extracted from high resolution MRI images (A-C). Asphericity coefficient was calculated as an AL/ED ratio; circumference and area are increased in the mutants. Two-way ANOVA post hoc Tukey test was used; ***P<0.001, values are mean ± SEM of 4 animals per age and genotype. (TIF) [file pone.0129518.s002.tif]

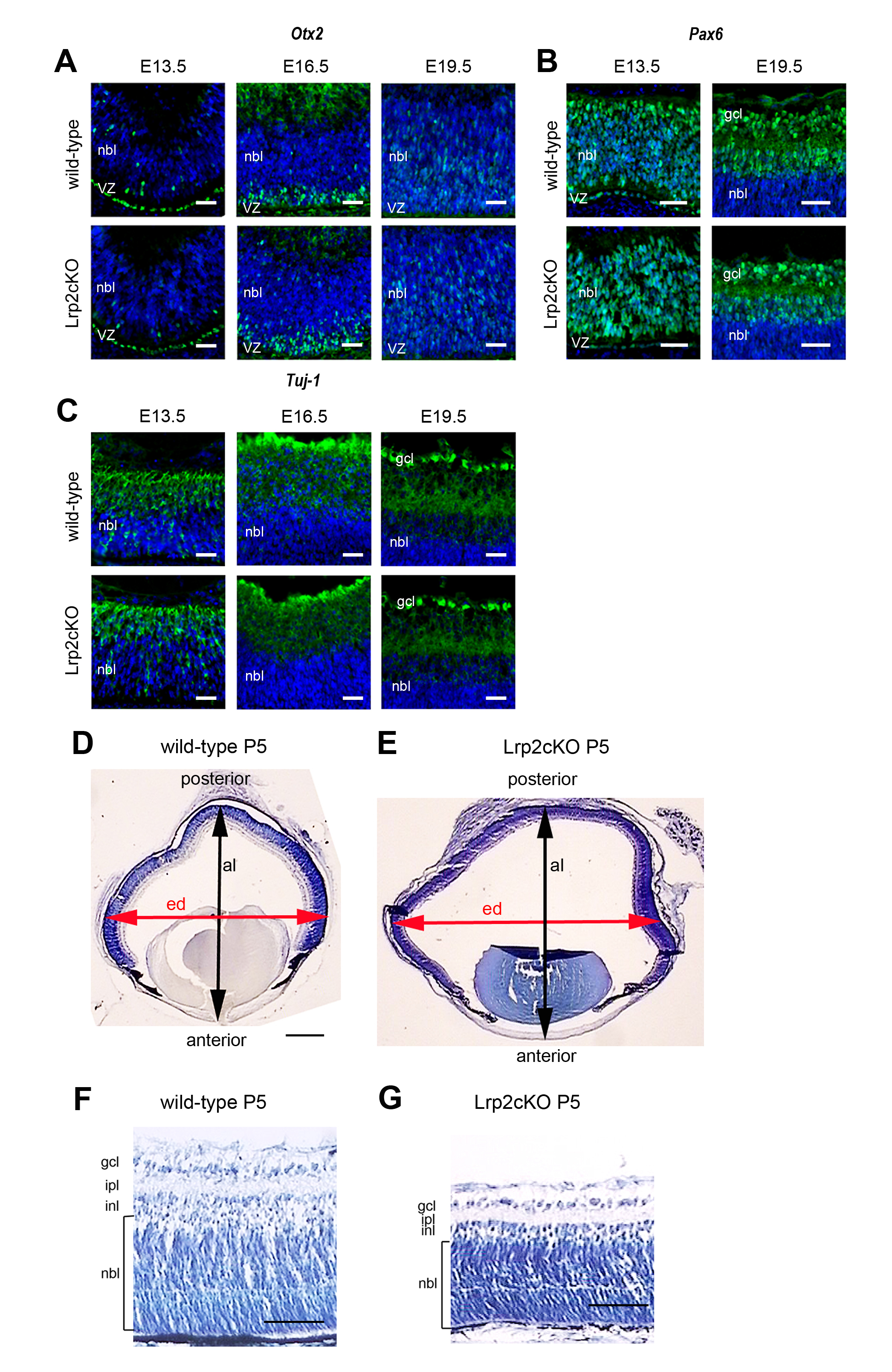

Supplement: S3 Fig — Retinal cryosections of control and mutant eyes between E13.5 and E19.5 show similar distribution of OTX2 (A), PAX6 (B) and TUJ-1 (C) in the developing retina. Nissl staining on sagittal cryosections of control and mutant eyes at P5 showing increased AL (black double-headed arrow) and equatorial diameter (red double-headed arrow) in the mutants (D, E). Retinal thickness is reduced in the mutants (F, G). Scale bars: 25 μm A-C; 300 μm in D, E, 90 μm in F, G. (TIF) [file pone.0129518.s003.tif]

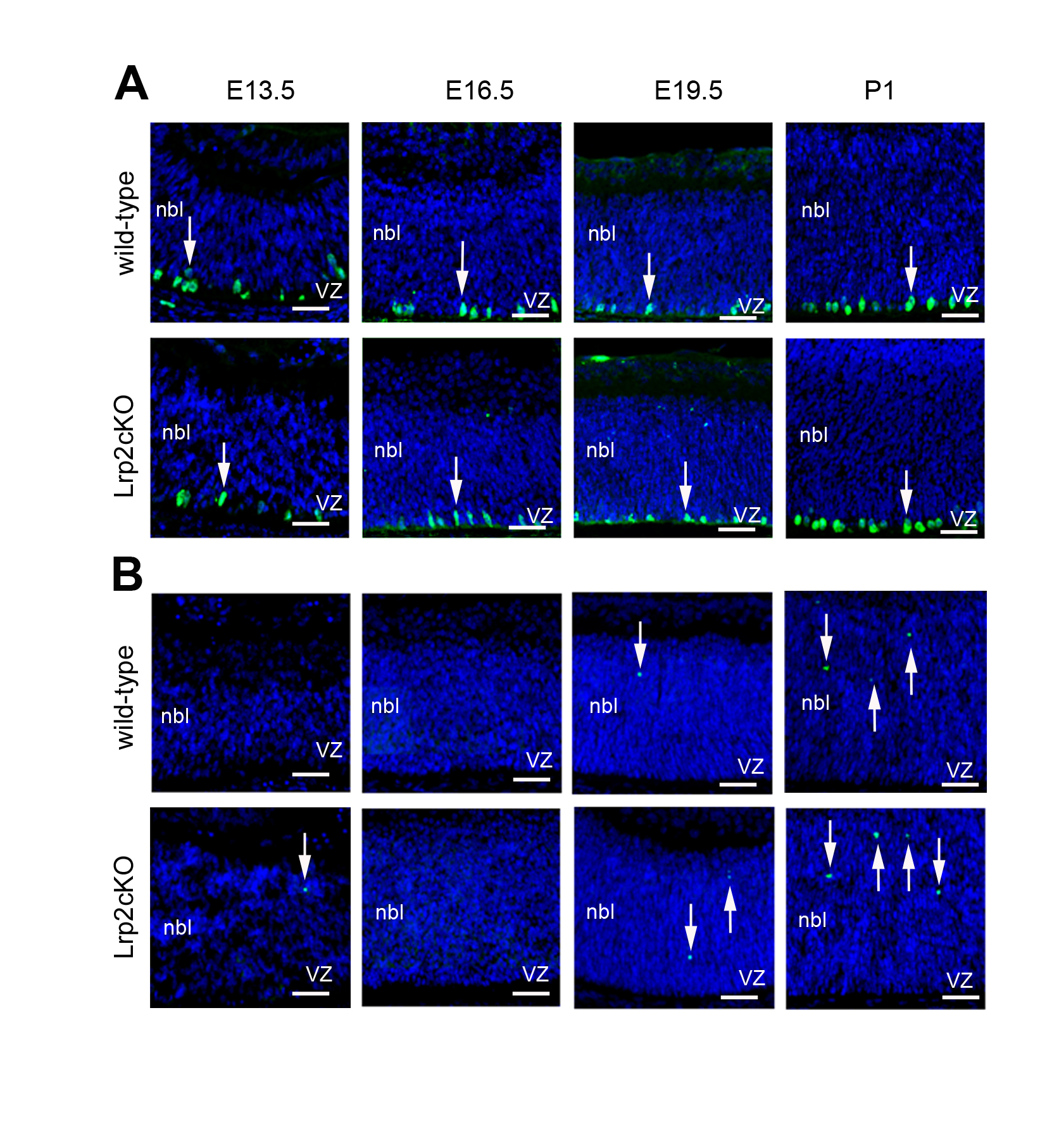

Supplement: S4 Fig — The cell proliferation PH3 immunostaining (green) is similarly distributed in the ventricular zone (vz) of control and mutant eyes at the ages indicated (A). TUNEL + cells (white arrows) are occasionally found in control and mutant retinas at the ages indicated (B). Scale bars: 25 μm in A, B. (TIF) [file pone.0129518.s004.tif]

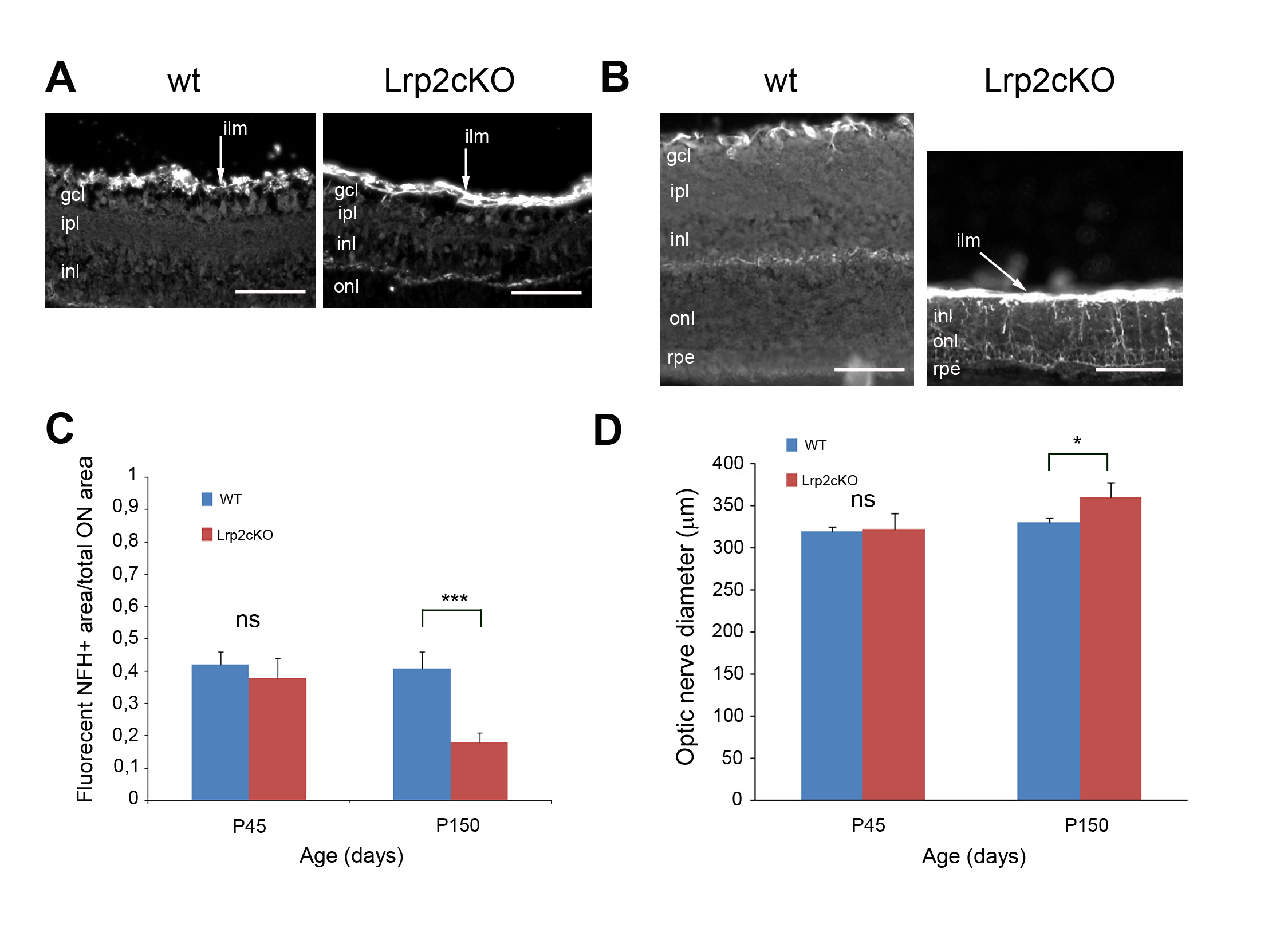

Supplement: S5 Fig — GFAP staining on retinal cryosections of control and mutant retinas at P10 (A) and P90 (B). The signal is increased in the astrocytes (arrows) of the mutants. Neurofilament Heavy Chain (NFH) staining is used to count the optic nerve axons at the ages indicated (C). Despite the significant reduction of NFH+ fibers (55%) observed at P150 in the mutant optic nerve, the diameter of the mutant optic nerve is slightly higher than that of the control (D). Comparisons are made between the two groups, age matched controls and Lrp2 FoxG1.cre-KO by two-tailed, unpaired t test. C: P45, p = 0.099; P150, p*** = 1.07E-6<0.001. D: P45, p = 0.23; P150, p* = 0.022<0.05. Values are mean ± SEM of 4 animals per age and genotype. Scale bars: 50 μm in A; 30 μm in B. (TIF) [file pone.0129518.s005.tif]

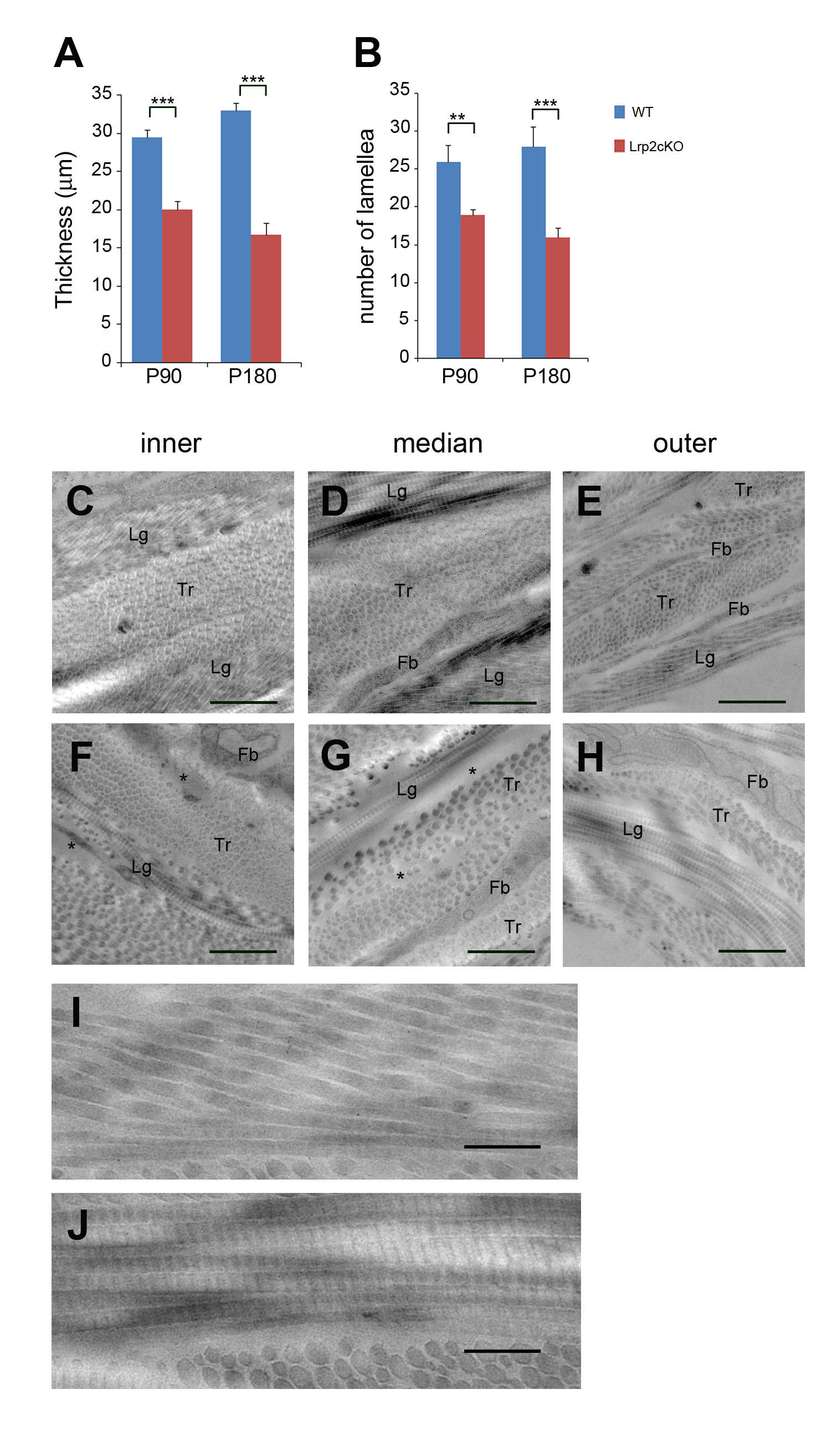

Supplement: S6 Fig — Reduced posterior sclera thickness (A) is associated with a reduction in the number of lamellae across the entire mutant sclera (B) at the ages indicated. Transmission electron microscopy reveals collagen architecture in the inner, middle and outer layers of the posterior sclera in control (C, D, E, I) and mutant (F, G, H, J) eyes at P90. Decreased interweaving, abnormal collagen packing of the mutant fibrils and fibril-free spaces, asterisks in (F, G), are seen throughout the mutant retina. Comparisons are made between the two groups, age matched controls and Lrp2 FoxG1.cre-KO by two-tailed, unpaired t test. A: P90, p*** = 0.000255<0.001; P180, p*** = 3.99E-4<0.001. B: P90, p** = 0.0018<0.01; P180, p*** = 0.00027<0.001. Values are mean ± SEM of 3 animals per age and genotype. Scale bars: 1 μm in C-H; 600 nm in I, J. (TIF) [file pone.0129518.s006.tif]
