# Supplementary material for: Foxg1-Cre Mediated Lrp2 Inactivation in the Developing Mouse Neural Retina, Ciliary and Retinal Pigment Epithelia Models Congenital High Myopia
Source: PLoS One. 2015 Jun 24;10(6):e0129518. doi: 10.1371/journal.pone.0129518 (PMC4480972; doi:10.1371/journal.pone.0129518)
Supplement: S1 Table — (DOCX) [file pone.0129518.s007.docx]

| Antigen | target | Dilution | Company | Cat num |
| --- | --- | --- | --- | --- |
| Megalin/lrp2 | neuroepithelium | 1/2,000 | lab |  |
| Brn3a | RGC | 1/125 | Millipore | MAB1585 |
| Tuj-1 | Postmitotic cell | 1/250 | Millipore | MAB1637 |
| Hsp70 | this study | 1/200 | StressMarq | SMC-114C/D |
| Aquaporin4 | Astrocyte | 1/250 | Millipore | AB3594 |
| Cleaved caspase 3 | Apoptotic cell | 1/200 | BD pharmingen | 557035 |
| GFAP | astrocyte | 1/500 | Dako | Z0334 |
| Phospho histone 3 | Mitotic cell | 1/200 | Millipore | MABE13 |
| Otx2 | Retinal progenitor and RPE | 1/250 | Millipore | AB9566 |
| Pax6 | Neural retina | 1/100 | Covance | PRB-278P |
| PKCα | bipolar cell | 1/1,000 | Santa Cruz | SC-208 |
| 200kDa neurofilament | RGC axon | 1/1,000 | Abcam | Ab8135 |
